# The Association between Glucose 6-Phosphate Dehydrogenase Deficiency and Attention Deficit/Hyperactivity Disorder

**DOI:** 10.3390/nu15234948

**Published:** 2023-11-29

**Authors:** Eugene Merzon, Eli Magen, Shai Ashkenazi, Abraham Weizman, Iris Manor, Beth Krone, Ilan Green, Avivit Golan-Cohen, Shlomo Vinker, Stephen V. Faraone, Ariel Israel

**Affiliations:** 1 Adelson School of Medicine, Ariel University, Ariel 40776, Israel; shaias@ariel.ac.il; 2 Leumit Health Services, Tel Aviv 64738, Israel; igreen@leumit.co.il (I.G.); agolanchoen@leumit.co.il (A.G.-C.); svinker@leumit.co.il (S.V.); aisrael@leumit.co.il (A.I.); 3 Department of Medicine A, Assuta Ashdod University Hospital, Faculty of Health Sciences, Ben Gurion University, Beer Sheba 84990, Israel; allergologycom@gmail.com; 4 ADHD Unit, Geha Mental Health Center, Petah Tikva 49100, Israel; weizmana@gmail.com (A.W.); dr.iris.manor@gmail.com (I.M.); 5 Department of Psychiatry, Faculty of Medicine, Tel Aviv University, Tel Aviv 69978, Israel; 6 Laboratory of Molecular and Biological Psychiatry, Sackler Faculty of Medicine, Tel Aviv University, Tel Aviv 69978, Israel; 7 Icahn School of Medicine at Mount Sinai, New York, NY 10029, USA; beth.krone@mssm.edu; 8 Department of Family Medicine, Faculty of Medicine, Tel Aviv University, Tel Aviv 69978, Israel; 9 Department of Psychiatry, Norton College of Medicine, SUNY Upstate Medical University, Syracuse, NY 13210, USA; sfaraone@childpsychresearch.org; 10 Department of Epidemiology and Disease Prevention, Faculty of Medicine, Tel Aviv University, Tel Aviv 69978, Israel

**Keywords:** attention deficit hyperactivity disorder (ADHD), glucose 6-phosphate dehydrogenase (G6PD), neurodevelopmental disorder, somatic, mental health, oxidative stress, neuroinflammation

## Abstract

Background: Glucose-6-phosphate dehydrogenase (G6PD) deficiency, impacting 4.9% of the population and more prevalent in Mediterranean communities, is a common enzymopathy with potential relevance to Attention Deficit/Hyperactivity Disorder (ADHD). This study investigated this association. Methods: The clinical characteristics of 7473 G6PD-deficient patients and 29,892 matched case–controls (selected at a 1:4 ratio) from a cohort of 1,031,354 within the Leumit Health Services database were analyzed using Fisher’s exact test for categorical variables and the Mann–Whitney U test for continuous variables. Results: In total, 68.7% were male. The mean duration of follow-up was 14.3 ± 6.2 years at a mean age of 29.2 ± 22.3 years. G6PD deficiency was associated with an increased risk of being diagnosed with ADHD (Odds Ratio (OR) = 1.16 [95% CI, 1.08–1.25], *p* < 0.001), seeking care from adult neurologists (OR = 1.30 [95% CI, 1.22–1.38], *p* < 0.001), and consulting adult psychiatrists (OR = 1.12 [95% CI, 1.01–1.24], *p* = 0.048). The use of stimulant medications among G6PD-deficient individuals was 17% higher for the methylphenidate class of drugs (OR = 1.17 [95% CI, 1.08, 1.27], *p* < 0.001), and there was a 16% elevated risk for amphetamine use (OR = 1.16 [95% CI, 1.03, 1.37], *p* = 0.047). Conclusions: G6PD deficiency signals an increased risk of ADHD diagnosis, more severe presentations of ADHD and a greater need for psychiatric medications to treat ADHD.

## 1. Introduction

Attention deficit/hyperactivity disorder (ADHD) is a common neurodevelopmental disorder that affects approximately 5–10% of children and 2–5% of adults worldwide. While ADHD is highly heritable, its etiology is complex, and there is strong evidence for an interplay of inflammatory processes with genetic risk in the development of ADHD [1]. Perhaps the most compelling evidence has come from longitudinal studies showing that maternal inflammatory processes confer greater risk for later ADHD diagnoses among youth with higher ADHD polygenic risk scores [1]. Youth with ADHD continue to express inflammatory markers well beyond the perinatal period [2], however, suggesting that the pathophysiology of ADHD is an ongoing process of epigenetic risk rather than a longitudinal result of prenatal insults. There is evidence that inflammatory markers may correlate to temperament [3] and amplify the weight of other bio-behavioral factors, such as sleep hygiene, in predicting the development of ADHD [4]. These associations vary with age and ADHD presentation [5] due to environmental exposures or age-related health factors. Two emerging lines of study link microglial dysregulation [6] and T cell abnormality [7], respectively, as actors in these associations.

Another intriguing but under-studied line of research also implicates the immune–inflammatory pathways’ association with oxidative stress in the development of ADHD [2,8,9,10]. Oxidative stress is a state of chemical imbalance with effects on the innate immune system that impact the growth and death of cells throughout the body, including the central nervous system [2].

Glucose-6-phosphate dehydrogenase (G6PD) is an enzyme that facilitates the production of nicotinamide adenine dinucleotide phosphate (NADPH) and glutathione (GSH), both of which play essential roles in maintaining the balance of oxidation–reduction in the body [11,12]. G6PD deficiency results in lower levels of GSH and therefore greater oxidative stress. G6PD deficiency, an X-linked genetic disorder requiring sex-specific detection methods [13], is the most common enzymopathy of humans. Globally, it affects approximately 4.9% of individuals and is characterized by hemolysis and the oxidative cell death of leukocytes, myocytes, and other immunological actors [12]. While G6PD deficiency may confer protection against Plasmodium falciparum malaria, common antimalarial treatments may induce the oxidative cell death process [13]. Other triggers include infections, certain foods or other drugs [12]. The most common presentation of G6PD in primary care is food-induced and known as favism, which is a hemolytic reaction to the consumption of fava beans, also known as broad beans. In pediatrics, however, severe hemolysis hyperbilirubinemia and jaundice due to G6PD deficiency in newborns can lead to hearing deficits, behavior disorders, and long-lasting neurologic damage [14]. G6PD mutations are common among geographically defined populations, including those originating in the Mediterranean Basin and other parts of the Middle East [15], and some East Asian [16] and African populations. The metabolic changes linked to G6PD deficiency may result in an elevated production of reactive oxygen species (ROS) and an imbalance in the antioxidant system [11,17], setting off a molecular cascade resulting in impaired astrocyte function, neuron death [18], and damage to DNA. ROS can activate astrocytes and microglia, which may produce proinflammatory chemokines and cytokines at high levels, contributing to neuro-inflammation and adversely impacting brain development [19,20].

**Figure 1:**
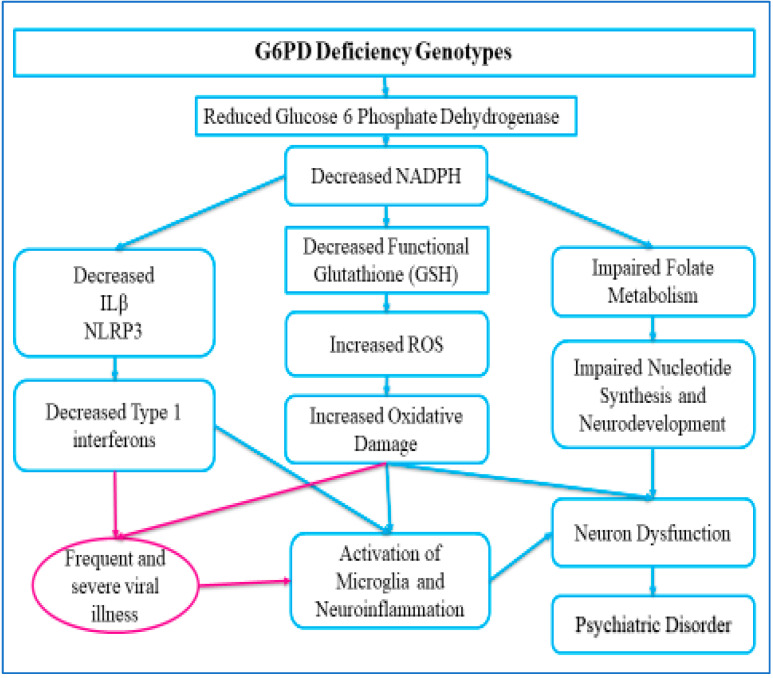
G6PD deficiency pathways to ADHD. G6PD = Glucose-6-phosphate dehydrogenase; ILꞵ = interleukin 1 beta, which amplifies immune response; NLRP3 = NLR family pyrin domain containing 3, recognizes pathogens; NADPH = nicotinamide adenine dinucleotide phosphate, a coenzyme required for cellular processes.

In 2010, Ghanizadeh et al. suggest that G6PD deficiency may be a predisposing factor for ADHD [21]. In their study, a small clinical sample of parents of children diagnosed with ADHD were questioned regarding their child’s history of a G6PD deficiency diagnosis. Out of 27 parents, 3 (11.1%) reported that their children were previously diagnosed with G6PD deficiency. Another prospective preliminary study found low levels of GSH and elevated oxidative stress among individuals with ADHD [8]. The association between ADHD, oxidative stress, and neuroinflammation arises from factors such as the imbalance between oxidants and antioxidants, catecholaminergic dysregulation, medications utilized for treatment, and genetic and environmental factors [8,9,10], as illustrated in Figure 1.

Some studies suggest an association between ADHD and a subset of patients with lower vagal tone and higher oxidative stress [22]. These studies demonstrated an association between low vagal tone and ADHD, suggesting that the connection between the vagal pathway and neurodevelopmental disorders is mediated through microbiota. However, a larger body of research implicates a link between ADHD and oxidative stress, as evidenced by the possible beneficial effect of antioxidant substances such as omega-3 fatty acids [23] and N-acetylcysteine [24] in subpopulations of youth with ADHD.

Newer investigations have revealed links between ADHD and a range of infectious disorders [25,26,27], autoimmune disorders [28], and neuroinflammation [29,30,31,32,33]. Other studies have highlighted the importance of social determinants of health in shaping health outcomes, and oxidative stress is one pathway through which social functioning can directly impact physical and mental health, with NADPH-oxidase-mediated oxidative stress initiating a biological cascade resulting in anxiety, depression, and possibly ADHD [34]. Understanding the interplay between social stressors, oxidative stress, and neuroinflammation during child development may help in identifying new avenues for mitigating the adverse consequences of these genetically conferred conditions.

The main objective of the present study was to evaluate the possible association between G6PD deficiency and ADHD, in light of our understanding of neuro-inflammation as a contributing factor, in a large-scale population-based study.

## 2. Materials and Methods

### 2.1. Methods

We performed a retrospective population-based cohort study using electronic health records (EHRs) from Leumit Health Services (LHS), a large Health Maintenance Organization (HMO) in Israel. This study was approved by the LHS institutional review board (LEU 0005-22, accessed on 16 February 2022).

### 2.2. Study Population

Data were retrieved from the LHS data warehouse. The study population was extracted from a pool of over 1,000,000 individuals who had been members of LHS for at least two years up to the data extraction date. LHS maintains a computerized database continuously updated with information related to subjects’ demographics, medical diagnoses, laboratory tests, hospitalizations, and medication prescriptions.

### 2.3. Study Design

In this cohort study, two groups of individuals were compared. The first group comprised individuals who have a confirmed diagnosis of G6PD deficiency or whose G6PD activity levels were tested at LHS and yielded a measurement below 4 U/g Hg.

An algorithm was utilized to randomly choose individuals without G6PD deficiency as a control cohort. The algorithm required precise matching for variables such as gender, age, socioeconomic status (SES), a category based on geographic area, ethnic group (General, Jewish Ultra-Orthodox, and Arabs), and year of first documented visit in the LHS EHR system compared to individuals with G6PD deficiency. The ratio of controls to individuals with G6PD deficiency was set at 4:1. Individuals with G6PD deficiency for whom the matching algorithm could not identify adequate controls with a precise match in variables were excluded from the analysis.

Demographic and clinical data, ICD-9 diagnosis records, the most recent recorded body mass index (BMI), blood pressure (BP) measurements, and smoking habits were obtained from the LHS electronic database. In addition, the last available results for a specific set of laboratory tests conducted by LHS laboratory facilities were also extracted.

### 2.4. Definitions

ADHD was diagnosed according to the Israeli Ministry of Health criteria, following the international evaluation requirements: the diagnosing physician must be a senior physician specializing in the ADHD field (child or adult psychiatrists, child or adult neurologists, or pediatricians and family physicians with certified ADHD training), and the diagnosis was established according to the Diagnostic and Statistical Manual (DSM-4 or 5, depending on the year of the diagnosis) criteria [35].

SES was defined according to the child’s home address, using the Israeli Central Bureau of Statistics classification of 20 subgroups. Classifications 1–7 were defined as low SES, 8–13 as medium SES, and 14–20 as high SES.

The likelihood of confounding was reduced by strictly matching for essential variables (age, gender, ethnic group, and SES) between the control group and the cases.

Descriptive statistics were used to describe the baseline cohort demographic and clinical characteristics, and the statistical association between categorical variables was assessed using Fisher’s exact test. Continuous variables were compared using the Mann–Whitney U test. Data extraction was performed using programs developed by the Leumit Research Institute in Python, Pandas, and SQL. Statistics were computed on de-identified data using R statistical software, version 4.0.4.

## 3. Results

From a group of N = 1,031,354 individuals who had been insured for at least two years by LHS with a documented medical history in the EHR, we could identify 7827 subjects with a G6PD deficiency diagnosis. The strict matching procedure resulted in selecting 7473 subjects with G6PD deficiency and 29,892 control subjects (at a ratio of 4:1 controls) with very similar gender, age, SES category, ethnic group, and year of first recorded visit to LHS. In total, 354 G6PD-deficient individuals were excluded due to insufficient matching controls. The socio-demographic characteristics of the study groups are shown in Table 1. The matched variables, which included age distribution, age categories, gender, ethnic group, and SES, were highly similar, with many parameters being identical between the two groups. These similarities, with no statistically significant differences, suggest that the matching was very strict. In both groups, the mean age of the patients was 29.2 ± 22.3 years, with 68.7% being male. The average follow-up time was 14.3 ± 6.2 years.

Clinical characteristics for the two groups are presented in Table 2. ADHD rates were significantly higher among G6PD-deficient individuals, with 1040/7473 (13.9%) cases vs. 3650/29,892 (12.2%) among the matched controls; Odds Ratio (OR) = 1.16 [95% CI, 1.08–1.25], *p* < 0.001. G6PD-deficient individuals were characterized by higher rates of being non-smokers (OR = 1.12 [95% CI, 1.07–1.19], *p* < 0.001). Having a diagnosis of diabetes mellitus (DM) was negatively associated with G6PD deficiency (OR = 0.85 [95% CI 0.76–0.95], *p* < 0.001). Being obese (BMI ≥ 30) also has a negative association with G6PD deficiency (OR = 0.92 [95% CI, 0.85–0.99, *p* = 0.042]).

### 3.1. Physician Visits

As evidence of the increased diagnoses of ADHD among G6PD-deficient individuals, the rates of physician visits were significantly higher among subjects with G6PD deficiency than the matched controls (Table 3). A significant difference was found in the number of visits to adult neurologists and adult psychiatrists (OR = 1.30 [95% CI, 1.22–1.38], *p* < 0.001; OR = 1.12 [95% CI, 1.01–1.24], *p* = 0.048, respectively).

### 3.2. Stimulant Agents Prescribed

The rates of stimulants prescribed to the subjects with G6PD deficiency were significantly higher compared to those prescribed to the subjects without G6PD deficiency (for methylphenidate use, OR = 1.17 [95% CI, 1.08–1.27], *p* < 0.001, and for amphetamine use, OR = 1.16 [95% CI, 1.03–1.37], *p* = 0.047) (Table 3).

The rates of physician visits and stimulant agent use among ADHD patients with and without G6PD deficiency.

### 3.3. Healthcare Utilization

We compared the healthcare resource utilization of the 1040 patients diagnosed with ADHD in the G6PD-deficient group with the 3650 patients diagnosed with ADHD in the matched non-G6PD-deficient group. We observed a higher number of visits to neurologists per ADHD patient in the G6PD-deficient group than in the non-G6PD-deficient group (3.57 vs. 3.05, *p* < 0.001). We also observed significantly higher rates of the use of stimulants among ADHD patients with G6PD deficiency as compared to non-G6PD-deficient patients (OR = 1.19, [95% CI, 1.00–1.42], *p* = 0.044), However, we did not observe significant differences in the drug classes prescribed between the two groups.

## 4. Discussion

This large-population cohort study showed that G6PD-deficient individuals have a significantly higher rate of ADHD diagnosis compared to matched controls. It also demonstrates the substantially higher rate of visits to adult neurologists and adult psychiatrists. As far as we know, this is the first large-scale population-based epidemiological study to evaluate the association between G6PD deficiency and ADHD. This finding is in line with the association between ADHD and inflammatory somatic conditions [36,37,38], between ADHD and infectious diseases [25,26,27] and between G6PD deficiency and COVID-19 infection and immune-related diseases [39]. G6PD-deficient patients, similar to individuals with ADHD, exhibit a higher incidence of infectious diseases worldwide, indicating a vulnerability to infections [40,41]. Exposure to infectious conditions and certain foods or medicines among G6PD-deficient subjects affect RBCs, leading to hemolysis associated with neurological and psychiatric problems.

We suggest several possible mechanistic explanations for the association between ADHD and G6PD deficiency.

The presence of oxidative stress: individuals with G6PD deficiency are at risk of hemolytic anemia in states of oxidative stress, which can result from infection, exposure to medications, and certain foods. In healthy conditions, G6PD is a crucial enzyme in the pathway of dinucleotide phosphate (NADPH) creation, which maintains the supply of reduced glutathione in the cells used to mop up free radicals that cause oxidative damage. Oxidative stress is also one of the proposed etiological mechanisms of ADHD, although it is not yet clear enough [2,10]. It is possible that the oxidative stress seen in G6PD deficiency results in subtle damage to the developing brain [10]. Such a putative insult combined with genetic vulnerability and adverse environmental factors may contribute to the evolvement of ADHD symptomatology. It seems that in specific conditions, G6PD deficiency increases the levels of free radicals and causes oxidative damage in the brain and periphery.

NADPH is required for the synthesis of neurotransmitters such as dopamine and serotonin [42]. These neurotransmitters are known to be involved in regulating attention, mood, and behavior, and imbalances in their levels have been implicated in the development of ADHD. Since G6PD plays a vital role in the production of NADPH, it is possible that G6PD deficiency could disrupt the balance between these neurotransmitters and contribute to the development of ADHD [43,44].

The dysregulation of reactive oxygen species (ROS) represents a crucial mechanism that can contribute to a heightened and more severe inflammatory response in individuals, thereby increasing the symptomatic nature of infection diseases [39,45,46]. Studies have demonstrated that individuals with ADHD exhibited higher rates of infections including COVID-19 [25,26] with higher rates of severity [29,47] and complications [27]. Similarly, it was shown that G6PD-deficient individuals have a higher incidence of infectious diseases, indicating a predisposition to infections [40,41,45,46,48]. Our previous study showed that G6PD-deficient individuals had a higher risk of COVID-19 infection, of COVID-19 hospitalization, and of developing long-COVID [46]. Taken together, these findings suggest that there is a possible association between G6PD deficiency and ADHD mediated by a shared increased risk of infection and inflammation. Several studies showed a significant association between individuals with ADHD and autoimmune disorders [36], supporting the hypothesis of an immunological vulnerability in ADHD patients. There is also an association between G6PD deficiency and immune-system-associated diseases [39]. Immune dysregulation may play a role in the pathogenesis of autoimmune and infectious diseases in both disorders, presumably through dysregulated NADPH homeostasis and ROS imbalances.

A comparison of healthcare resource utilization shows that patients diagnosed with ADHD and G6PD deficiency sought specialists more often and purchased more stimulants when compared with ADHD patients who are not G6PD-deficient, suggesting a possibly increased severity of ADHD symptoms among these G6PD-deficient patients. Thus, it could be that apart from a common etiological mechanism, G6PD deficiency could be an additive factor that increases the severity of already existing ADHD.

We observed a substantially higher rate of visits to adult neurologists and adult psychiatrists by patients with ADHD and G6PD deficiency. Several explanations are plausible. First, it is currently acceptable that although the diagnosis of ADHD is typically made in childhood or adolescence, patients diagnosed with ADHD often require lifelong follow-up and treatment. This continuity of care leads individuals with ADHD to seek psychiatric services from adult specialists, especially during the transition from adolescence to adulthood.

Secondly, the substantially higher rate of visits to adult neurologists and adult psychiatrists of our patients with ADHD could be explained by the specific healthcare landscape and access to child and adolescent neurology and psychiatric services in Israel. Most child neurologists and psychiatrists work in hospital settings, and we might have had a limited ability to include them in our analysis. According to the Israel Child and Adolescent Psychiatric Association, the number of child and adolescent psychiatrists is limited, with a notable gap between the psychiatric care needed and the care actually received. Consequently, many individuals under the age of 19 years, including those with ADHD, end up receiving treatment from adult psychiatrists, which can also explain the observed higher rates of adult neurologist and psychiatrist visits.

Some studies described a positive association between G6PD deficiency and a risk of diabetes mellitus (DM) [49,50], but a case–control study from Sardinia, Italy, showed no statistically significant negative association [51]. We assume that a negative association with having a diagnosis of DM observed in our study may be explained by the fact that G6PD deficiency can lead to alterations in HbA1c levels that are not reflective of real glucose levels. Previous research has shown that individuals with G6PD deficiency have significantly lower HbA1c levels than those without the deficiency, even when their glucose levels are similar. This can result in significant underdiagnoses of DM [52,53].

A population-representative Chinese birth cohort showed that G6PD deficiency was associated with a lower BMI, which line aligns with our data.

Our findings showed an association between G6PD deficiency and a lower BMI, consistent with the finding from a population-representative Chinese birth cohort [54]. Future studies are needed to explore this association further and to be able to investigate interventions that can prevent the emergence of ADHD or attenuate its manifestations in individuals with G6PD deficiency.

### Strengths and Limitations

The major strength of the present study is its extensive scope, population-based nature, and utilization of real-world data. Furthermore, the study used advanced digital data to analyze various demographic, clinical, and laboratory factors that may influence the risk of ADHD. As far as the researchers know, this is the first large epidemiological study to thoroughly examine the association between G6PD deficiency and ADHD using a considerably large nationwide database.

The main limitation of this study pertains to its observational and retrospective design, which means that some unidentified potential confounders may have impacted the outcomes. Therefore, we report our conclusions using the terminology of association and probability rather than causality.

Since our sample primarily consisted of Middle Eastern and Mediterranean populations who identify as Caucasians, and as G6PD deficiency is also of a higher prevalence among East Asian and African populations, these findings may not represent the entire scope of the G6PD community, and the results may not apply to other ethnic groups. However, our study encourages further research to replicate our results in different populations and in a prospective design to identify causality and underlying mechanisms.

## 5. Conclusions

Both ADHD and G6PD deficiency are genetically conferred developmental disorders that begin in infancy and co-travel within the population. More research is required to determine their interplay across the lifespan. It is possible that the oxidative stress seen in G6PD deficiency results in subtle damage to the developing brain which, when combined with genetic vulnerability or compounded by adverse environmental factors, may contribute to the development of ADHD. Oxidative stress remains a proposed etiological mechanism of ADHD, although there is not enough evidence yet to state with certainty that it is causative. More research into the etiological mechanisms of ADHD through a lens of oxidative stress is warranted.

This population-based nationwide study demonstrated a significant association between G6PD deficiency and ADHD. Physicians and allied healthcare providers need to be aware of this potential association for several reasons. First, G6PD deficiency is a sex-linked disorder that manifests with the greatest severity among males, but a large population of females experience symptoms below the thresholds customarily associated with disease among males. Diagnosing G6PD deficiency among women remains a challenge due to sex-based presentation, and different laboratory methods are required for determining females with the disorder, but there may be substantial neurological consequence to undiagnosed and untreated disease processes. Awareness of the cognitive and behavioral consequences of these disorders should prompt practitioners to look for the sex disparities in G6PD and in ADHD presentations among their patients. Second, regardless of the sex of the patient, there are substantial neuropsychiatric consequences of G6PD that may be preventable with early diagnosis and prevention strategies. Finally, if new etiological mechanisms are identified, treatments for individuals with G6PD and ADHD may be improved. Currently, there is evidence that stimulant treatment with methylphenidate alters oxidative stress pathways, though studies conflict as to whether treatment is associated with improved [55] or more dysregulated [56] processes. Further, there may be complementary treatments to augment and improve medication outcomes in this population [57].

## Figures and Tables

**Table 1:** Demographic characteristics of the G6PD deficient group and matched control group.

		G6PD-Deficient Group	Matched Controls	*p*	Odds Ratio [95% CI]
N		7473	29,892		
Gender, N (%)	Female	2327 (31.1%)	9308 (31.1%)	1	1.00 [0.94–1.06]
	Male	5146 (68.9%)	20,584 (68.9%)	1	1.00 [0.94–1.06]
Age in years M(SD)		29.2 (22.3)	29.2 (22.3)	0.984	1.00 [0.94–1.06]
Age in years N (%)	0–2	378 (5.06%)	1512 (5.06%)	1	1.00 [0.91–1.10]
	3–9	1331 (17.81%)	5324 (17.81%)	1	1.00 [0.93–1.07]
	10–18	1268 (16.97%)	5072 (16.97%)	1	1.00 [0.93–1.08]
	19–29	1355 (18.13%)	5420 (18.13%)	1	1.00 [0.93–1.07]
	30–39	1053 (14.09%)	4212 (14.09%)	1	1.00 [0.92–1.08]
	40–49	623 (8.34%)	2488 (8.32%)	1	1.00 [0.91–1.10]
	50–59	531 (7.11%)	2128 (7.12%)	1	1.00 [0.91–1.10]
	60–69	460 (6.16%)	1841 (6.16%)	1	1.00 [0.90–1.11]
	70–79	260 (3.48%)	1039 (3.48%)	1	1.00 [0.86–1.16]
	80–89	152 (2.03%)	608 (2.03%)	1	1.00 [0.80–1.24]
	≥90	62 (0.83%)	248 (0.83%)	1	1.00 [0.42–2.07]
Ethnic group N (%)	Arab	729 (9.76%)	2916 (9.76%)	1	1.00 [0.91–1.09]
	General	5096 (68.19%)	20,384 (68.19%)	1	1.00 [0.94–1.06]
	Ultra-Orthodox	1648 (22.05%)	6592 (22.05%)	1	1.00 [0.94–1.07]
Region N (%)	Center	2160 (28.9%)	8408 (28.13%)	0.212	1.04 [0.98–1.10]
	Jerusalem	2330 (31.18%)	7239 (24.22%)	0.001	1.42 [1.34–1.51]
	North	1466 (19.62%)	5406 (18.09%)	0.002	1.11 [1.03–1.18]
	South	1517 (20.3%)	8835 (29.56%)	0.001	0.61 [0.57–0.65]
SES	M (SD)	9.2 (3.6)	9.2 (3.6)	1	1.00 [0.91–1.09]
Missing	N (%)	652 (9.2%)	2608 (9.2%)	1	1.00 [0.91–1.09]
SES N (%)	Low	1457(34.5%)	9828(34.5%)	1	1.00 [0.94–1.07]
	Low-Middle	1357 (19.1%)	5428 (19.1%)	1	1.00 [0.94–1.07]
	Middle-High	1564 (22.0%)	6256 (22.0%)	1	1.00 [0.94–1.07]
	High	1092 (15.3%)	4368 (15.3%)	1	1.00 [0.93–1.08]
	~Missing~	652 (9.2%)	2608 (9.2%)	1	1.00 [0.91–1.09]

**Table 2:** Clinical characteristics of the G6PD-deficient group and matched control group.

		G6PD Deficient Group	Matched Controls	*p*	Odds Ratio [95% CI]
N		7473	29,892		
Smoking status	Non-smoker	3505 (76.93%)	13,073 (74.77%)	0.001	1.12 [1.07–1.19]
	Past smoker	83 (1.8%)	305 (1.8%)	0.489	1.09 [0.84–1.40]
	Smoker	968 (21.25%)	4096 (23.43%)	0.186	0.94 [0.87–1.01]
	Missing	2917 (39.03%)	12,407 (41.51%)	0.001	0.90 [0.85–0.95]
BMI (kg/m^2^)		23.33 (6.14)	23.45 (6.32)	0.104	
Obesity, n (%)		915 (13.6%)	3934 (15.0%)	0.042	0.92 [0.85–0.99]
Hypertension, n (%)		804 (10.8%)	3365 (11.3%)	0.971	0.97 [0.89–1.05]
Diabetes Mellitus, n (%)		418 (5.59%)	1952 (6.53%)	0.035	0.85 [0.76–0.95]
ADHD, n (%)		1040 (13.9%)	3650 (12.2%)	0.001	1.16 [1.08–1.25]

**Table 3:** Physician visits and the use of stimulants among the G6PD-deficient group and matched control group.

	G6PD-Deficient Group	Matched Controls	*p*	Odds Ratio [95% CI]
N	7473	29,892		
Adult Neurologist Visits, n (%)	1746 (23.4%)	5687 (19.1%)	0.001	1.30 [1.22–1.38]
Child Neurologist Visits, n (%)	604 (8.1%)	2253 (7.5%)	0.073	1.08 [0.98–1.19]
Adult Psychiatrist Visits, n (%)	514 (6.9%)	1846 (6.2%)	0.048	1.12 [1.01–1.24]
Child Psychiatrist Visits, n (%)	161 (2.2%)	615 (2.1%)	0.586	1.05 [0.87–1.25]
ADHD-trained PCP Visits, n (%)	115 (1.5%)	410 (1.3%)	0.054	1.12 [0.98–1.39]
Methylphenidate Use, n (%)	929 (12.4%)	3233 (10.8%)	0.001	1.17 [1.08–1.27]
Amphetamine, n (%)	195 (2.6%)	675 (2.2%)	0.041	1.16 [1.03–1.37]

## Data Availability

Data are contained within the article.

## References

[1] Takahashi N., Nishimura T., Harada T., Okumura A., Iwabuchi T., Rahman S., Kuwabara H., Takagai S., Usui N., Makinodan M. (2023). Interaction of genetic liability for attention deficit hyperactivity disorder (ADHD) and perinatal inflammation contributes to ADHD symptoms in children. Brain Behav. Immun. Health.

[2] Koç S., Güler E.M., Derin S., Gültekin F., Aktaş S. (2023). Oxidative and Inflammatory Parameters in Children and Adolescents With ADHD. J. Atten. Disord..

[3] O’Connor T.G., Scheible K., Sefair A.V., Gilchrist M., Blackmore E.R., Winter M.A., Gunnar M.R., Wyman C., Carnahan J., Moynihan J.A. (2017). Immune and neuroendocrine correlates of temperament in infancy. Dev. Psychopathol..

[4] Morales-Muñoz I., Upthegrove R., Lawrence K., Thayakaran R., Kooij S., Gregory A.M., Marwaha S. (2023). The role of inflammation in the prospective associations between early childhood sleep problems and ADHD at 10 years: Findings from a UK birth cohort study. J. Child. Psychol. Psychiatry.

[5] Corominas-Roso M., Armario A., Palomar G., Corrales M., Carrasco J., Richarte V., Ferrer R., Casas M., Ramos-Quiroga J. (2017). IL-6 and TNF-α in unmedicated adults with ADHD: Relationship to cortisol awakening response. Psychoneuroendocrinology.

[6] Mordelt A., de Witte L.D. (2023). Microglia-mediated synaptic pruning as a key deficit in neurodevelopmental disorders: Hype or hope?. Curr. Opin. Neurobiol..

[7] Çetin F.H., Uçaryılmaz H., Uçar H.N., Artaç H., Güler H.A., Duran S.A., Kılınç K., Türkoğlu S. (2022). Regulatory T cells in children with attention deficit hyperactivity disorder: A case-control study. J. Neuroimmunol..

[8] Joseph N., Zhang-James Y., Perl A., Faraone S.V. (2015). Oxidative Stress and ADHD: A Meta-Analysis. J. Atten. Disord..

[9] Leffa D.T., Torres I.L.S., Rohde L.A. (2018). A Review on the Role of Inflammation in Attention-Deficit/Hyperactivity Disorder. Neuroimmunomodulation.

[10] Sa-Carneiro F., Calhau C., Coelho R., Figueiredo-Braga M. (2020). Putative shared mechanisms in autism spectrum disorders and attention deficit hyperactivity disorder, a systematic review of the role of oxidative stress. Acta Neurobiol. Exp..

[11] Luzzatto L., Ally M., Notaro R. (2020). Glucose-6-phosphate dehydrogenase deficiency. Blood.

[12] Georgakouli K., Fatouros I.G., Draganidis D., Papanikolaou K., Tsimeas P., Deli C.K., Jamurtas A.Z. (2019). Exercise in Glucose-6-Phosphate Dehydrogenase Deficiency: Harmful or Harmless? A Narrative Review. Oxidative Med. Cell. Longev..

[13] Peters A.L., Van Noorden C.J. (2009). Glucose-6-phosphate dehydrogenase deficiency and malaria: Cytochemical detection of heterozygous G6PD deficiency in women. J. Histochem. Cytochem..

[14] Allahverdiyev A.M., Bagirova M., Elcicek S., Koc R.C., Ates S.C., Baydar S.Y., Yaman S., Abamor E.S., Oztel O.N. (2012). Glucose-6-Phosphate Dehydrogenase Deficiency and Malaria: A Method to Detect Primaquine-Induced Hemolysis in vitro. Dehydrogenases.

[15] Abu Omar R., Algur N., Megged O., Hammerman C., Kaplan M. (2015). Glucose-6-Phosphate Dehydrogenase Screening in Israel-Arab and Palestinian-Arab Neonates. J. Pediatr..

[16] Wei H., Wang C., Huang W., He L., Liu Y., Huang H., Chen W., Zheng Y., Xu G., Lin L. (2022). Simultaneous detection of G6PD mutations using SNPscan in a multiethnic minority area of Southwestern China. Front. Genet..

[17] Le Pichon J.B., Riordan S.M., Watchko J., Shapiro S.M. (2017). The Neurological Sequelae of Neonatal Hyperbilirubinemia: Definitions, Diagnosis and Treatment of the Kernicterus Spectrum Disorders (KSDs). Curr. Pediatr. Rev..

[18] Corona J.C., Duchen M.R. (2015). Impaired mitochondrial homeostasis and neurodegeneration: Towards new therapeutic targets?. J. Bioenerg. Biomembr..

[19] Moniczewski A., Gawlik M., Smaga I., Niedzielska E., Krzek J., Przegaliński E., Pera J., Filip M. (2015). Oxidative stress as an etiological factor and a potential treatment target of psychiatric disorders. Part 1. Chemical aspects and biological sources of oxidative stress in the brain. Pharmacol. Rep..

[20] Smaga I., Niedzielska E., Gawlik M., Moniczewski A., Krzek J., Przegaliński E., Pera J., Filip M. (2015). Oxidative stress as an etiological factor and a potential treatment target of psychiatric disorders. Part 2. Depression, anxiety, schizophrenia and autism. Pharmacol. Rep..

[21] Ghanizadeh A., Namazi M.R., Davami M.H. (2010). G6PD Deficiency as a predisposing factor for attention deficit/hyperactivity disorder: A hypothesis. Arch. Med. Res..

[22] Petra A.I., Panagiotidou S., Hatziagelaki E., Stewart J.M., Conti P., Theoharides T.C. (2015). Gut-Microbiota-Brain Axis and Its Effect on Neuropsychiatric Disorders with Suspected Immune Dysregulation. Clin. Ther..

[23] Chang J.P., Su K.P., Mondelli V., Pariante C.M. (2018). Omega-3 Polyunsaturated Fatty Acids in Youths with Attention Deficit Hyperactivity Disorder: A Systematic Review and Meta-Analysis of Clinical Trials and Biological Studies. Neuropsychopharmacology.

[24] Garcia R.J., Francis L., Dawood M., Lai Z., Faraone S.V., Perl A. (2013). Attention deficit and hyperactivity disorder scores are elevated and respond to N-acetylcysteine treatment in patients with systemic lupus erythematosus. Arthritis Rheum..

[25] Merzon E., Gutbir Y., Vinker S., Cohen A.G., Horwitz D., Ashkenazi S., Sadaka Y. (2021). Early Childhood Shigellosis and Attention Deficit Hyperactivity Disorder: A Population-Based Cohort Study with a Prolonged Follow-up. J. Atten. Disord..

[26] Merzon E., Israel A., Ashkenazi S., Rotem A., Schneider T., Faraone S.V., Biederman J., Green I., Golan-Cohen A., Vinker S. (2023). Attention-Deficit/Hyperactivity Disorder Is Associated with Increased Rates of Childhood Infectious Diseases: A Population-Based Case-Control Study. J. Am. Acad. Child. Adolesc. Psychiatry.

[27] Merzon E., Weiss M., Krone B., Cohen S., Ilani G., Vinker S., Cohen-Golan A., Green I., Israel A., Schneider T. (2022). Clinical and Socio-Demographic Variables Associated with the Diagnosis of Long COVID Syndrome in Youth: A Population-Based Study. Int. J. Environ. Res. Public. Health.

[28] He H., Yu Y., Liew Z., Gissler M., László K.D., Valdimarsdóttir U.A., Zhang J., Li F., Li J. (2022). Association of Maternal Autoimmune Diseases with Risk of Mental Disorders in Offspring in Denmark. JAMA Netw. Open.

[29] Merzon E., Weiss M.D., Cortese S., Rotem A., Schneider T., Craig S.G., Vinker S., Cohen A.G., Green I., Ashkenazi S. (2022). The Association between ADHD and the Severity of COVID-19 Infection. J. Atten. Disord..

[30] Tylee D.S., Lee Y.K., Wendt F.R., Pathak G.A., Levey D.F., De Angelis F., Gelernter J., Polimanti R. (2022). An Atlas of Genetic Correlations and Genetically Informed Associations Linking Psychiatric and Immune-Related Phenotypes. JAMA Psychiatry.

[31] Vázquez-González D., Carreón-Trujillo S., Alvarez-Arellano L., Abarca-Merlin D.M., Domínguez-López P., Salazar-García M., Corona J.C. (2023). A Potential Role for Neuroinflammation in ADHD. Adv. Exp. Med. Biol..

[32] Lauden A., Geishin A., Merzon E., Korobeinikov A., Green I., Golan-Cohen A., Vinker S., Manor I., Weizman A., Magen E. (2021). Higher rates of allergies, autoimmune diseases and low-grade inflammation markers in treatment-resistant major depression. Brain Behav. Immun. Health.

[33] Dunn G.A., Nigg J.T., Sullivan E.L. (2019). Neuroinflammation as a risk factor for attention deficit hyperactivity disorder. Pharmacol. Biochem. Behav..

[34] Li H., Xia N. (2020). The role of oxidative stress in cardiovascular disease caused by social isolation and loneliness. Redox Biol..

[35] American Psychiatric Association (2022). Diagnostic and Statistical Manual of Mental Disorders.

[36] Faraone S.V., Banaschewski T., Coghill D., Zheng Y., Biederman J., Bellgrove M.A., Newcorn J.H., Gignac M., Al Saud N.M., Manor I. (2021). The World Federation of ADHD International Consensus Statement: 208 Evidence-based conclusions about the disorder. Neurosci. Biobehav. Rev..

[37] Vinker-Shuster M., Golan-Cohen A., Merhasin I., Merzon E. (2019). Attention-Deficit Hyperactivity Disorder in Pediatric Patients with Type 1 Diabetes Mellitus: Clinical Outcomes and Diabetes Control. J. Dev. Behav. Pediatr..

[38] Vinker-Shuster M., Eldor R., Green I., Golan-Cohen A., Manor I., Merzon E. (2022). Glycemic Control and Diabetes Related Complications in Adults with Type 1 Diabetes Mellitus and ADHD. J. Atten. Disord..

[39] Israel A., Schäffer A.A., Berkovitch M., Ozeri D.J., Merzon E., Green I., Golan-Cohen A., Ruppin E., Vinker S., Magen E. (2023). Glucose-6-phosphate dehydrogenase deficiency and long-term risk of immune-related disorders. Front. Immunol..

[40] Alrahmany D., Omar A.F., Al-Maqbali S.R., Harb G., Ghazi I.M. (2022). Infections in G6PD-Deficient Hospitalized Patients-Prevalence, Risk Factors, and Related Mortality. Antibiotics.

[41] Alrahmany D., Omar A.F., Hafez W., Albaloshi S., Harb G., Ghazi I.M. (2023). Infections in Glucose-6-Phosphate Dehydrogenase G6PD-Deficient Patients; Predictors for Infection-Related Mortalities and Treatment Outcomes. Antibiotics.

[42] Lopez-Lopez A., Villar-Cheda B., Quijano A., Garrido-Gil P., Garcia-Garrote M., Díaz-Ruiz C., Muñoz A., Labandeira-Garcia J.L. (2021). NADPH-Oxidase, Rho-Kinase and Autophagy Mediate the (Pro)renin-Induced Pro-Inflammatory Microglial Response and Enhancement of Dopaminergic Neuron Death. Antioxidants.

[43] Jiang L., Chen S., Chu C., Wang S., Oyarzabal E., Wilson B., Sanders V., Xie K., Wang Q., Hong J. (2015). A novel role of microglial NADPH oxidase in mediating extra-synaptic function of norepinephrine in regulating brain immune homeostasis. Glia.

[44] Begieneman M.P.V., ter Horst E.N., Rijvers L., Meinster E., Leen R., Pankras J.E., Fritz J., Kubat B., Musters R.J.P., van Kuilenburg A.B.P. (2016). Dopamine induces lipid accumulation, NADPH oxidase-related oxidative stress, and a proinflammatory status of the plasma membrane in H9c2 cells. Am. J. Physiol. Heart Circ. Physiol..

[45] Mondal A., Mukherjee S., Dar W., Upadhyay P., Ranganathan A., Pati S., Singh S. (2022). G6PD deficiency: Imbalance of functional dichotomy contributing to the severity of COVID-19. Future Microbiol..

[46] Israel A., Berkovitch M., Merzon E., Golan-Cohen A., Green I., Ruppin E., Vinker S., Magen E. (2023). Glucose-6-Phosphate Dehydrogenase Deficiency and Coronavirus Disease 2019. Clin. Infect. Dis..

[47] Sadaka Y., Freedman J., Ashkenazi S., Vinker S., Golan-Cohen A., Green I., Israel A., Eran A., Merzon E. (2021). The Effect of Antibiotic Treatment of Early Childhood Shigellosis on Long-Term Prevalence of Attention Deficit/Hyperactivity Disorder. Children.

[48] Wang Q., Xu R., Volkow N.D. (2020). Increased risk of COVID-19 infection and mortality in people with mental disorders: Analysis from electronic health records in the United States. World Psychiatry.

[49] Santana M.S., Brito M.A.M., Sampaio V.S., Monteiro W.M., Costa M.R.F., Alecrim M.G.C., Lacerda M.V.G. (2014). High frequency of diabetes and impaired fasting glucose in patients with glucose-6-phosphate dehydrogenase deficiency in the Western brazilian Amazon. Am. J. Trop. Med. Hyg..

[50] Heymann A.D., Cohen Y., Chodick G. (2012). Glucose-6-phosphate dehydrogenase deficiency and type 2 diabetes. Diabetes Care.

[51] Pinna A., Contini E.L., Carru C., Solinas G. (2013). Glucose-6-phosphate dehydrogenase deficiency and diabetes mellitus with severe retinal complications in a Sardinian population, Italy. Int. J. Med. Sci..

[52] Kweka B., Lyimo E., Jeremiah K., Filteau S., Rehman A.M., Friis H., Manjurano A., Faurholt-Jepsen D., Krogh-Madsen R., PrayGod G. (2020). Influence of hemoglobinopathies and glucose-6-phosphate dehydrogenase deficiency on diagnosis of diabetes by HbA1c among Tanzanian adults with and without HIV: A cross-sectional study. PLoS ONE.

[53] Leong A., Lim V.J.Y., Wang C., Chai J.-F., Dorajoo R., Heng C.-K., van Dam R.M., Koh W.-P., Yuan J.-M., Jonas J.B. (2020). Association of G6PD variants with hemoglobin A1c and impact on diabetes diagnosis in East Asian individuals. BMJ Open Diabetes Res. Care.

[54] Kwok M.K., Leung C.M., Schooling C.M. (2016). Glucose-6-Phosphate Dehydrogenase Deficiency and Physical and Mental Health until Adolescence. PLoS ONE.

[55] Sinningen K., Emons B., Böhme P., Juckel G., Hanusch B., Beckmann B., Tsikas D., Lücke T. (2023). l-Arginine/nitric oxide pathway and oxidative stress in adults with ADHD: Effects of methylphenidate treatment. Nitric Oxide.

[56] Miniksar D.Y., Cansız M.A., Göçmen A.Y., Kılıç M., Miniksar Ö.H. (2023). The Effect of Drug Use, Body Mass Index and Blood Pressure on Oxidative Stress Levels in Children and Adolescents with Attention Deficit and Hyperactivity Disorder. Clin. Psychopharmacol. Neurosci..

[57] Sangouni A.A., Mirhosseini H., Hosseinzadeh M. (2022). Effect of vitamin D supplementation on brain waves, behavioral performance, nitric oxide, malondialdehyde, and high-sensitivity C-reactive protein in children with attention deficit/hyperactivity disorder: Study protocol for a randomized clinical trial. Trials.

